# A Highly Sensitive and Group-Specific Enzyme-Linked Immunosorbent Assay (ELISA) for the Detection of AFB_1_ in Agriculture and Aquiculture Products

**DOI:** 10.3390/molecules29102280

**Published:** 2024-05-12

**Authors:** Junlin Cao, Ting Wang, Kang Wu, Fengjie Zhou, Yuze Feng, Jianguo Li, Anping Deng

**Affiliations:** 1 The Key Lab of Health Chemistry and Molecular Diagnosis of Suzhou, College of Chemistry, Chemical Engineering and Materials Science, Soochow University, Renai Road 199, Suzhou 215123, China; cjl9808@163.com (J.C.); 13383897519@163.com (T.W.); qq574929936@163.com (Y.F.); 2 School of Biology & Basic Medical Science, Soochow University, Renai Road 199, Suzhou 215123, China; 3 Suzhou Agricultural Products Safety and Quality Inspection Center, Wuzhong Road 1399, Suzhou 215000, China; zfj3732380@gmail.com

**Keywords:** aflatoxins, AFB_1_, enzyme-linked immunosorbent assay (ELISA), monoclonal antibody (mAb), agricultural and aquiculture products, total evaluation

## Abstract

Aflatoxins (AFs) including AFB_1_, AFB_2_, AFG_1_ and AFG_2_ are widely found in agriculture products, and AFB_1_ is considered one of the most toxic and harmful mycotoxins. Herein, a highly sensitive (at the pg mL^−1^ level) and group-specific enzyme-linked immunosorbent assay (ELISA) for the detection of AFB_1_ in agricultural and aquiculture products was developed. The AFB_1_ derivative containing a carboxylic group was synthesized and covalently linked to bovine serum albumin (BSA). The AFB_1_-BSA conjugate was used as an immunogen to immunize mice. A high-quality monoclonal antibody (mAb) against AFB_1_ was produced by hybridoma technology, and the mAb-based ELISA for AFB_1_ was established. IC_50_ and limit of detection (LOD) of the ELISA for AFB_1_ were 90 pg mL^−1^ and 18 pg mL^−1^, respectively. The cross-reactivities (CRs) of the assay with AFB_2_, AFG_1_, and AFG_2_ were 23.6%, 42.5%, and 1.9%, respectively, revealing some degree of group specificity. Corn flour, wheat flour, and crab roe samples spiked with different contents of AFB_1_ were subjected to ELISA procedures. The recoveries and relative standard deviation (RSD) of the ELISA for AFB_1_ in spiked samples were 78.3–116.6% and 1.49–13.21% (*n* = 3), respectively. Wheat flour samples spiked with the mixed AF (AFB_1_, AFB_2_, AFG_1_, AFG_2_) standard solution were measured by ELISA and LC-MS/MS simultaneously. It was demonstrated that the proposed ELISA can be used as a screening method for evaluation of AFs (AFB_1_, AFB_2_, AFG_1_, AFG_2_) in wheat flour samples.

## 1. Introduction

Aflatoxins (AFs), the highly toxic secondary metabolites produced by *Aspergillus* flavus and *Aspergillus* parasiticus fungi, are present in a wide range of food and feed commodities, and are assumed significant because of their deleterious effects on human beings, poultry, and livestock [1,2,3]. There are up to 18 types of AFs, but only 6 of them (AFB_1_, AFB_2_, AFG_1_, AFG_2_, AFM_1_, and AFM_2_) are regulated for food control [4]. The first four are the major pollutants in agriculture products, while AFM_1_ and AFM_2_, which are mainly found in milk, are generated by some mammals such as cows after consuming feed contaminated with AFB_1_ or AFB_2_ [5]. Among the six AFs above, AFB_1_ is considered the most widely distributed, toxic, and harmful [6]. Various agriculture and food products such as corn, wheat, peanuts, soybeans, cooking oil, alcoholic beverages, and poultry feeds are susceptible to AFB_1_ contamination [7]. AFB_1_ has been classified as a human carcinogen (Group 1) by the International Agency for Research on Cancer (IARC) due to its carcinogenic, mutagenic, and teratogenic potential [8]. Because of the risk of the presence of AFs in foods, many countries have implemented corresponding regulations. The European Union has established that the maximum residue level (MRL) of total AFs (AFB_1_, AFB_2_, AFG_1_, and AFG_2_) in food should not exceed 10 μg kg^−1^. In addition, AFB_1_ has an individual MRL of 5 μg kg^−1^ in foods for human consumption and 0.1 μg kg^−1^ in products for infants [9]. In China, the MRL of AFB_1_ is also specified, including for peanut oil and corn oil (20 µg kg^−1^), wheat, barley, and their products (5 µg kg^−1^), corn and corn products (20 µg kg^−1^), and cooked nuts (5 µg kg^−1^), and is 0.5 µg kg^−1^ in infants [10]. Hence, reliable and sensitive analytical methods for trace AFB_1_ detection are of paramount importance.

The main analytical methods used for the detection of AFB_1_ in agriculture products are liquid chromatography–tandem mass spectrometry (LC-MS/MS) and high-performance liquid chromatography with fluorescence detection (HPLC-FLD) [11,12,13]. These chromatographic methods have high sensitivity, selectivity, and accuracy, but have limitations related to the requirement for qualified and skilled personnel, expensive equipment, time-consuming processes, and large sample volumes involved.

Immunoassays, particularly enzyme-linked immunosorbent assays (ELISAs), are sensitive and specific analytical methods based on the interaction between the antibody and antigen [14,15]. In addition, ELISAs display other advantages such as rapid performance, simple sample preparation, high throughput, and low cost for sample testing. In recent decades, many immunoassays, including chemiluminescence immunoassay [16], time-resolved fluorescence immunoassay [17], electrochemical immunosensors [18], and immunochromatographic assay [19], have been reported for the detection of AFB_1_. Furthermore, many direct competitive (dc)- or indirect competitive (ic)-ELISAs based on polyclonal antibodies (pAbs) or monoclonal antibodies (mAbs) for detecting AFB_1_ have been developed (Table 1) [20,21,22,23,24,25,26,27,28,29,30,31,32,33]. However, establishing a sensitive immunoassay is mainly dependent upon the quality of antibody. Therefore, the research on producing a high-quality antibody against AFB_1_ has continued in the last thirty years. Furthermore, it is desirable for ELISA to evaluate the total AFs (AFB_1_, AFB_2_, AFG_1_, and AFG_2_) in agricultural products. In addition, AFB_1_ may contaminate fish feed, and also accumulate in aquiculture products [34]. Currently, however, the samples for AFB_1_ testing are mainly focused on agriculture products, and there are few samples involved.

Herein, a highly sensitive and group-specific mAb-based ELISA for the detection of AFB_1_ in agriculture and aquiculture products was developed. Using the keto carbonyl group that exists in the AFB_1_ molecule, AFB_1_ was reacted with o-carboxymethyl hydroxylamine (CMO) to form an oxime, and the carboxylic group in the oxime molecule was used for covalent linking to bovine serum albumin (BSA). The AFB_1_-BSA conjugate was used as an immunogen to immunize mice. After making great efforts, a mAb with the highest affinity binding for AFB_1_ was successfully produced by hybridoma technology, and the corresponding ELISA for AFB_1_ was established. To examine the accuracy and precision of the ELISA, agriculture and aquiculture samples including corn flour, wheat flour, and crab roe were spiked with AFB_1_, and the spiked samples were subjected to ELISA performance. Acceptable recoveries and relative standard deviation (RSD) of the ELISA for AFB_1_ in spiked samples were obtained. As the mAb also showed some degree of group specificity with other AFs, to verify the practicability of the ELISA for screening total AFs (AFB_1_, AFB_2_, AFG_1_, and AFG_2_) in agriculture products, wheat flour samples spiked with the mixed AF standard solution (AFB_1_, AFB_2_, AFG_1_, AFG_2_) were measured by ELISA and LC-MS/MS simultaneously.

## 2. Results and Discussion

### 2.1. Synthesis of Immunogen and Coating Antigen

**Figure 1:**
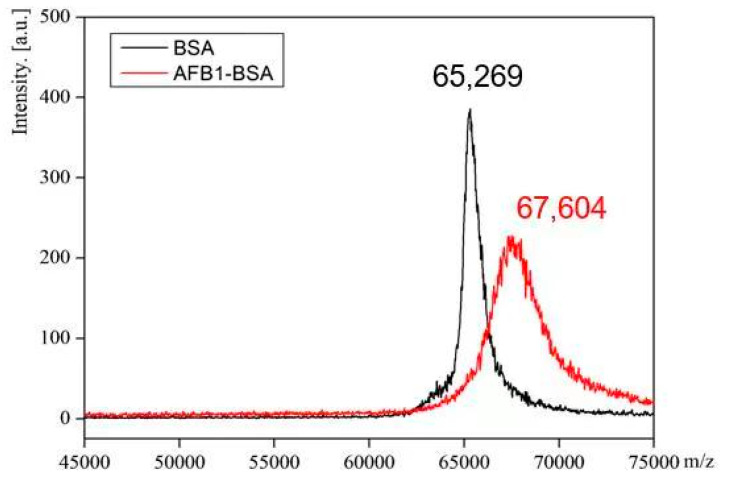
MALDI-TOF-MS spectra of BSA and AFB_1_-BSA. The *m*/*z* values of BSA and AFB_1_-BSA are 65,269 and 67,604, respectively.

AFB_1_ is a small molecular compound without sufficient immunogenicity; thus, to produce an antibody against AFB_1_, AFB_1_ should couple to a carrier protein to form an AFB_1_–protein conjugate. In this study, taking advantage of the keto carbonyl group in the AFB_1_ molecule, AFB_1_ was reacted with CMO to form an oxime molecule containing a carboxylic group at one end (Figure S1). Via the carboxylic group and by the carbodiimide method, the AFB_1_ derivative was covalently coupled to a carrier protein (BSA or OVA) to form an AFB_1_-BSA (or AFB_1_-OVA) conjugate. The coupling ratio of AFB_1_/BSA in AFB_1_-BSA conjugate was determined by MALDI-TOF-MS. The typical MALDI-TOF-MS spectra of BSA and AFB_1_–BSA are shown in Figure 1. It can be seen from Figure 1 that the molecular weights of BSA and AFB_1_-BSA are 65,269 and 67,604, respectively. As the molecular weight of AFB_1_-CMO is 385, and OH is lost through coupling, the coupling ratio of AFB_1_/BSA in AFB_1_-CMO-BSA is estimated to be (67,604 − 65,269)/(385 − 17) = 6.3, which indicates that in each BSA molecule, about 6.3 AFB_1_ molecules have been linked to the surface of BSA. The MALDI-TOF-MS spectra of OVA and AFB_1_–OVA are similar to those in Figure 1 and are not given herein. Similar calculation results show that the coupling ratio of AFB_1_/OVA in AFB_1_–OVA is 2.9. These results indicate that the AFB_1_ has been successfully coupled to the carrier proteins with the appropriate coupling ratios in AFB_1_–protein conjugates.

### 2.2. Preparation of mAb against AFB_1_

Six female Balb/c mice were immunized with AFB_1_–BSA. Seven days after the fifth immunization, the sera were collected and measured by ic-ELISA. Among the six immunized mice, the mouse with the highest titer and inhibition effect was selected for cell fusion. Ten days after cell fusion, five positive cells capable of secreting antibodies against AFB_1_ were selected by ic-ELISA using AFB_1_ as a competitor. Among them, the cells (6C6) displayed the highest sensitivity for AFB_1_; thus, AFB_1_ was selected for subcloning via the limiting dilution method. The clones (6C6) were expanded and cryopreserved in liquid nitrogen. The large-scale production of mAb was from nude mice ascites. 6C6 hybridoma cells were intraperitoneally injected into nude mice that were pre-injected with liquid paraffin 1 week before. Seven days later, the ascites from the nude mice were collected and purified by the protein G extraction column. The purified mAbs were stored at −20 °C in the presence of 50% glycerol.

### 2.3. Optimization of ELISA Conditions

In order to achieve high sensitivity, the ELISA conditions, including the dilution of the coating antigen, dilution of the antibody, and GaMIgG-HRP, were optimized based on two criteria, i.e., (1) to achieve an IC_50_ value as low as possible, and (2) to achieve an absorbance in the range of 0.8–1.5 absorption units for the zero standard concentration according to the Lambert–Beer law. The optimal ELISA conditions were selected by the checkerboard titration method, in which the coating antigen (AFB_1_–OVA, 1 mg mL^−1^) was diluted in the range of 1:500~1:10,000, while the dilutions of mAb (6C6) and GaMIgG-HRP were examined in the range of 1:5000~1:80,000 and 1:1000~1:20,000, respectively. The optimal ELISA conditions (e.g., antigen dilution at 1:1000, mAb dilution at 1:10,000, and HRP-GaMigG dilution at 1:5000) were finally obtained. 

### 2.4. Sensitivity of ELISA

**Figure 2:**
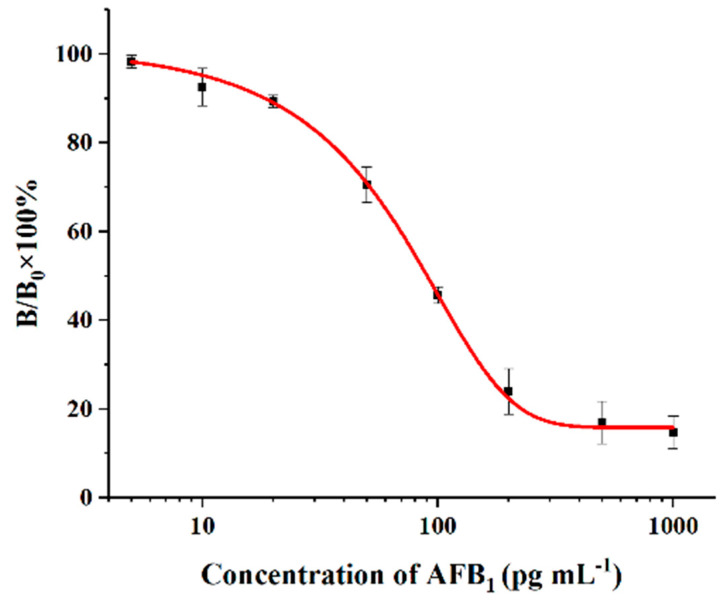
Standard curve of the ELISA for AFB_1_ detection under optimal experimental conditions. The bars represent the standard derivations of the values of B/B_0_ × 100% (*n* = 5). The IC_50_ was 90 pg mL^−1^, and the LOD was 18 pg mL^−1^.

Under optimal conditions, the AFB_1_ standard solutions were applied to ELISA procedures. The standard curve of the ELISA for AFB_1_ is shown in Figure 2. The average IC_50_ value calculated from the standard curves obtained from six consecutive measurements is 90 pg mL^−1^, while the limit of detection (LOD) of the ELISA based on three times the standard deviation (3 × SD) at zero concentration is 18 pg mL^−1^. 

Many mAb (or pAb)-based dc-ELISAs or ic-ELISAs for the detection of AFB1 in different samples have been developed in recent decades (Table 1) [20,21,22,23,24,25,26,27,28,29,30,31,32,33]. 

As shown in Table 1, the IC_50_ value achieved in our ELISA (in pg mL^−1^ level) is lower than most of those obtained in other ELISAs, which clearly demonstrates the high sensitivity of our ELISA. The high sensitivity of the newly produced mAb may be related to the coupling ratio in the immunogen (AFB_1_–BSA) and in the coating antigen (AFB_1_–OVA). An appropriate coupling ratio (6.3) of AFB1/BSA in the immunogen may be good for promoting a strong immune response in immunized mice, and thus for producing a high-quality antibody for AFB1. Furthermore, the relatively lower coupling ratio (2.9) of AFB1/OVA in the coating antigen may lead to a lower affinity binding force between the antibody and the coating antigen, which comparatively increases the affinity binding force between the antibody and AFB1, thus increasing the sensitivity further.

### 2.5. Specificity of the ELISA

The specificity of ELISA was evaluated by the cross-reactivity (CR) of mAb with the homologs of AFB_1_ (e.g., AFB_2_, AFG_1_, AFG_2_, AFM_1_, and AFM_2_) and five other mycotoxins (OTA, ZEN, DON, FB_1_, and patulin). The CR values of the mAb with these compounds are presented in Table 2. When the CR of the ELISA of AFB_1_ is considered to be 100%, it can be seen from Table 2 that there is no cross-reactivity (CR < 0.01%) of the mAb with OTA, ZEN, DON, FB_1_, and PAT, which demonstrates that other mycotoxins in the samples will not interfere with the detection of AFB_1_ measured by the proposed ELISA. Furthermore, from Table 2 it can be seen that there are some extents of the CR values (1.9~4.3%) of the mAb with AFB_1_ homologs. Specifically, for AFG_1_ and AFM_1_, which contain an olefinic bond in the furan ring, the CR values are 42.5% and 44.3%, respectively; while for AFG_2_ and AFM_2_ without the olefinic bond, the CR values are only 1.9% and 4.1%, respectively. These results indicate that the mAb exhibits, to some degree, group specificity with other AFs, and the olefinic bond plays an important role in CR values for AFB_1_ homologs. For AFB_2_, although there is no an olefinic bond in the furan ring, due to the high similarity in the molecular structure between AFB_1_ and AFB_2_, the CR of the mAb with AFB_2_ was as much as 23.6%.

### 2.6. Accuracy and Precision of the ELISA

The ELISA was further characterized by accuracy and precision, which were obtained from the spiking experiment. In this study, wheat flour, corn flour, and crab roe were spiked with AFB_1_ at the concentrations of 0~40 ng g^−1^. After extraction, the spiked and unspiked samples were subjected to ELISA procedures. As shown in Table 3, for three unspiked wheat flour, corn flour, and crab roe samples, the blank values were found to be 0.9, 0.21, and 0.37 ng g^−1^, which are lower than those of the MRL for human consumption set by EU and China. Furthermore, it is shown in Table 3 that the recoveries of the ELISA for AFB_1_ in spiked samples were 78.3–116.6%, with relative standard deviations (RSDs) of 1.49–13.21% (*n* = 3). The results reveal the high accuracy and precision of the ELISA for AFB_1_ in different samples. 

### 2.7. Analysis of Wheat Flour Sample Spiked with Total AFs

Normally, real agricultural samples may contain not only AFB_1_, but also other types of AFs. To verify the practicability of the proposed ELISA for screening total AFs (AFB_1_, AFB_2_, AFG_1_, and AFG_2_) in agriculture products, wheat flour samples were spiked with the mixed AF standard solution to give total spiked AF concentrations of 0, 12.5, 25, and 50 ng g^−1^. The spiked samples were measured by ELISA and LC-MS/MS simultaneously. The concentrations of individual AFs detected by LC-MS/MS are presented in Table S1, and the results of the mixed AF spiked samples measured by LC-MS/MS and ELISA are given in Table 4. From Table 4, it is seen that the concentrations measured by ELISA are about two times lower than those measured by LC-MS/MS, which is due to the fact that the CR values of the ELISA with AFB_2_, AFG_1_, and AFG_2_ are 23.6%, 42.5% and 1.9%, respectively. It is clear that the concentration measured by ELISA is mainly contributed by AFB_1_ (the CR of the ELISA with AFB_1_ is considered to be 100%), but AFB_2_, AFG_1_, and AFG_2_ are also contributors, dependent on their CR values. Thus, the proposed ELISA can be used as a screening method for total AFs (AFB_1_, AFB_2_, AFG_1_, and AFG_2_) in wheat flour samples. 

## 3. Materials and Methods

### 3.1. Reagents, Materials and Apparatus

AFB_1_, AFB_2_, AFG_1_, AFG_2_, AFM_1_, AFM_2_, ochratoxin A (OTA), zearalenone (ZEN), patulin (PAT), vomiting toxins (DON), and fumonisin B1 (FB_1_) were purchased from Tanmo Quality Inspection Technology Co., Ltd. (Beijing, China). O-carboxymethyl hydroxylamine (CMO) and pyridine were purchased from China National Pharmaceutical Group Shanghai Co., Ltd. (Shanghai, China). 1-(3-Dimethylaminopropyl)-3-ethylcarbodiimide hydrochloride (EDC), bovine serum albumin (BSA), ovalbumin (OVA), casein, RPMI-1640 medium, hypoxanthine aminopterin thymidine (HAT), hypoxanthine thymidine (HT), dimethylformamide (DMF), methanol, acetonitrile (ACN), dimethyl sulfoxide (DMSO), 3,3,3,5,5-tetramethylbenzidine (TMB), polyethylene glycol (PEG_1500_), and Freund’s complete and incomplete adjuvants were purchased from Sigma Co. (St. Louis, USA). The horseradish peroxidase labeled goat anti mouse IgG conjugate (HRP-GaMIgG) was purchased from Zhongshan Jinqiao Biotechnology Co., Ltd. (Beijing, China). Fetal bovine serum (FBS) was purchased from Life Technologies Co. (New York, NY, USA). The mouse SP2/0 myeloma cells were purchased from the cell bank of the Chinese Academy of Sciences (Shanghai, China). Balb/c mice (6–8 weeks old) were obtained from School of Medicine, Soochow University (Suzhou, China). Other chemicals were analytical grade.

Culture plates (24-well and 96-well cell) and cell culture flasks were purchased from Corning Inc. (Corning, NY, USA). The ultra-clean table was purchased from Haier Group (Qingdao, China). The CO_2_ incubator (HF 151 UV) was purchased from Heal Fore Development Ltd. (Shanghai, China). The spectrophotometer UV-2300 was purchased from Techcomp (Shanghai, China). The ELISA reader (Sunrise Remote/Touch Screen) and microtiter plate washer (M12/2R) were purchased from Columbus plus (Tecan, Grödig, Austria). The microtiter plate shaker (KJ-201C Oscillator) was bought from Kangjian Medical Apparatus, Co., Ltd. (Jiangsu, China). The deionized-RO water supply system (Dura 12FV) was purchased from The Lab Com. (Dover, DE, USA). MALDI-TOF-MS equipment (Micro Q-TOF) was purchased from Bruker Daltonics (Billerica, MA, USA).

### 3.2. Buffers and Solutions

In this study, the buffers and solutions used were as follows: (1) 0.05 mol L^−1^ carbonate buffer (pH 9.6) was used as a coating buffer; (2) coating antigen (AFB_1_-OVA conjugate) stock solution (1.0 mg mL^−1^) was prepared by dissolving 1.0 mg of AFB_1_-OVA in 1 mL of coating buffer; (3) assay buffer was 0.01 mol L^−1^ phosphate buffered saline (PBS, pH 7.4) containing 145 mmol L^−1^ NaCl; (4) washing buffer (PBST) was the assay buffer with 0.1% (*v*/*v*) Tween-20; (5) blocking solution was 1% casein in the assay buffer; (6) acetate buffer was 100 mmol L^−1^ sodium acetate acid buffer (pH 5.8); (7) substrate solution (TMB + H_2_O_2_) was prepared by adding 200 µL of 10 mg mL^−1^ TMB dissolved in DMSO, 3.5 µL of 30% H_2_O_2_ and 1 mL of acetate buffer to 20 mL of ultrapure water; (8) sulfuric acid (5%) was used to stop the enzymatic coloration; (9) AFB_1_ stock solution (1 mg mL^−1^) was prepared by dissolving 1.0 mg of AFB_1_ in 1.0 mL of methanol; (10) AFB_1_ working solution (1000 ng mL^−1^) was prepared by diluting AFB_1_ stock solution (1.0 mg mL^−1^) with the assay buffer; (11) AFB_1_ standard solutions with concentrations of 0, 10, 20, 50, 100, 200, 500, and 1000 pg mL^−1^ were prepared by diluting the AFB_1_ working solution (1000 ng mL^−1^) with the assay buffer.

### 3.3. Synthesis of Immunogen and Coating Antigen

AFB_1_ is a small molecular compound and does not have enough immunogenicity to stimulate the animal to produce antibodies. However, when AFB_1_ is coupled to BSA, the AFB_1_–BSA conjugate will obtain sufficient immunogenicity and can be used as an immunogen to immunize animals to produce antibodies. AFB_1_ has a keto carbonyl group, which can be easily reacted with CMO to form an oxime (Figure S1). Then, the carboxylic group in the oxime molecule can react with the amino group in BSA to form an amide bond. Briefly, 2 mg of AFB_1_ and 4 mg of CMO were added to 0.4 mL of pyridine, and the solution was stirred at 25 °C for 24 h. Thin-layer chromatography (TLC) was employed to check whether AFB_1_ had been fully reacted with CMO. After the reaction was complete, pyridine in the solution was evaporated by a nitrogen stream, and the dried residue containing the resultant oxime was obtained. AFB_1_–oxime was completely dissolved in 1.5 mL of DMF-water (1:1, *v*/*v*); then, 27.6 mg of EDC was added and mixed well in the dark. Subsequently, 2.8 mL of 0.13 mol L^−1^ NaHCO_3_ solution containing 14 mg of BSA was added. The solution was slightly stirred at 25 °C for 4 h, then 26 mg of EDC was added again and the reaction was continued for 24 h. The solution was transferred into a dialysis bag, and dialyzed at 4 °C for 3 days in a 0.01 mol L^−1^ PBS solution (pH 7.4) and for 1 day in ultrapure water. After dialysis, the dialyzed solution was centrifuged at 7000 r min^−1^ for 10 min, and the supernatant was lyophilized to a powder form. The synthesized AFB_1_–BSA conjugate was stored at −20 °C until use. Similarly, the AFB_1_–OVA conjugate was synthesized. The AFB_1_–BSA conjugate was used as the immunogen for mAb production, while the AFB_1_–OVA conjugate was used as the coating antigen for the establishment of ic-ELISA. 

### 3.4. Production of mAb against AFB_1_

The mAb against AFB_1_ was produced using the hybridoma technique. Six female Balb/c mice (6–8 weeks) were immunized with multiple subcutaneous injections of AFB_1_–BSA. In the first immunization, AFB_1_–BSA dissolved in physiological saline was emulsified with Freund’s complete adjuvant (1:1). Each mouse received an immunogen dose of 100 μg. The interval between two immunizations was 21 days. In the 2nd~4th booster immunizations, 100 μg of AFB_1_–BSA emulsified with incomplete Freund’s adjuvant (1:1) was given to each mouse. Seven days after the 3rd immunization, blood was collected from the tail of the immunized mice, and the titer and the inhibitory effect of the antisera were measured by ic-ELISA using AFB_1_–OVA as the coating antigen and AFB_1_ as the competitor, respectively. Three days before cell fusion, the mice were given the final booster injection intraperitoneally with 100 μg of AFB_1_–BSA without adjuvant. The mouse with the highest titer and inhibitory effect was selected for cell fusion. The mouse was sacrificed via cervical dislocation, and the spleen lymphocytes were evenly mixed with myeloma cells (SP2/0) at a 10:1 ratio and fused in 50% polyethylene glycol PEG4000 under aseptic conditions. The cell suspension was dispersed in 96-well cell culture plates containing RPMI-1640 medium, 20% fetal bovine serum, and 1% HAT (*v*/*v*), and cultured in an incubator at 37 °C with 5% CO_2_. The culture medium was changed every 3 days. After 10 days of cell culture, there were a large number of cells in the wells. The ic-ELISA was used to screen positive hybridoma cells that were able to specifically secrete antibodies against AFB_1_, and subcloning was performed using the limit dilution method. After subcloning, a large number of pure hybridoma cells capable of secreting antibodies were obtained. Stable mAb-producing clones were expended and cryopreserved in liquid nitrogen. Parts of the stable subclones were injected into the abdominal cavity of nude Balb/c mice to generate a large amount of mAb. 

Animal welfare and experimental procedures were strictly carried out according to recommendations in the Guide for the Care and Use of Laboratory Animals (23a) of the National Institutes of Health. All protocols were approved through the Institutional Animal Care and Use Committee (IACUC) of Soochow University. All efforts were made to minimize animal suffering and to reduce the number of animals used.

### 3.5. Procedures of ELISA

The ELISA procedures were as follows. AFB_1_–OVA diluted with coating buffer was added to a 96-well microplate (200 μL/well). The plate was placed at 4 °C overnight. After washing the plate three times with PBST, 280 μL/well of 1% casein was added to the plate to block the possible non-specific binding sites. After 1 h, the plate was washed with PBST three times. Then, the AFB_1_ standard solution (100 μL/well) and mAb solution (diluted with assay buffer, 100 μL/well) were added to the plate. The plate was slightly shaken at room temperature for 1 h. After washing the plate three times again, HRP-GaMIgG solution (200 μL/well) was added. The plate was placed at room temperature for 1 h, then washed with PBST three times. After washing the plate again, the enzymatic substrate solution (200 μL/each well) was added. The plate was slightly shaken for 15–20 min at room temperature. After washing, 80 μL/well of sulfuric acid (5%) was added to terminate the enzymatic reaction. The plate was slightly shaken for about 15 min at room temperature. The absorbances in the wells of the plate were measured with the ELISA reader at 450 nm. The standard curve of the ELISA for AFB_1_ was constructed by plotting the (B/B_0_) × 100% ~ log C_AFB1_, where B and B_0_ were the absorbances of the standard solution and zero concentration, respectively. From the standard curve, the IC_50_ (e.g., the AFB_1_ concentration that produces 50% signal inhibition) can be obtained. A lower IC_50_ value indicates higher sensitivity of the ELISA. The sample solution was subjected to the same ELISA procedures, and the concentration of AFB_1_ in the sample was calculated according to the standard curve obtained from the same plate.

### 3.6. Cross-Reactivity Testing

The specificity of the ELISA can be evaluated by cross-reactivity (CR) values. Besides AFB_1_, five AFs homologs (AFB_2_, AFG_1_, AFG_2_, AFM_1_, and AFM_2_) and other main mycotoxins including OTA, ZEN, DON, FB_1_, and PAT, were selected to test the CR (Table 2). The standard solutions of AFB_1_, AFB_2_, AFG_1_, and AFM_1_ were prepared in the concentration range of 10~1000 pg mL^−1^, while the standard solutions of AFG_2_ and AFM_2_ were in the range of 0.1~10 ng mL^−1^, and the standard solutions of OTA, ZEN, DON, FB_1_, and PAT were in the range of 10~10,000 ng mL^−1^. The standard solutions of the tested compounds were subjected to ELISA procedures. The values of IC_50_ can be obtained from the corresponding standard curves for every testing compound. The CR (%) was calculated based on the equation: CR (%) = (IC_50_ of AFB_1_/IC_50_ of the tested compound) × 100%.

### 3.7. Spiking Experiment

To examine the accuracy and precision of the ELISA, a spiking experiment was carried out. Wheat flour, corn flour, and crab roe were selected for the spiking experiment. Wheat flour and corn flour are normal food commodities, while crab is a popular aquiculture product enjoyed by the local people in the Suzhou area. It is possible that when the feed for crab farming is contaminated by AFB_1_, the toxic AFB_1_ can be accumulated in crab roe. Wheat flour, corn flour, and crab roe were purchased from the local market, and were spiked with AFB_1_ at the concentrations of 0~40 ng g^−1^. (Table 3). The spiked and unspiked samples were extracted, and then subjected to ELISA procedures. Briefly, to 4 glass tubes containing 2 g of wheat flour (or corn flour or homogenized crab roe), different volumes of AFB_1_ standard solution (1000 ng mL^−1^) were added (wheat flour: 0, 4, 10, and 20 μL; corn flour: 0, 20, 40, and 80 μL; crab roe: 0, 20, 40, and 80 μL). The tubes were intensively shaken at room temperature for 1 h, then 10 mL of ACN solution (ACN:water = 8:2, *v*/*v*) was added. The tubes were shaken at room temperature for 30 min, then centrifuged at 3000 r min^−1^ for 15 min. The supernatants were diluted with PBS at 1:10~1:200 to ensure the final concentration of AFB_1_ fell within the ELISA detection range. The diluted solutions were subjected to ELISA procedures. For each sample, three separate extractions were performed, and each sample was determined in triplicate. Recoveries were calculated based on the following equation: Recovery (%) = [(Conc. measured − blank)/Conc. spiked] × 100%.

### 3.8. Analysis of Wheat Flour Sample Spiked with Total AFs

Due to some degree of group specificity of the mAb with other AFs, to verify the proposed ELISA for screening total AFs (AFB_1_, AFB_2_, AFG_1_, and AFG_2_) in agriculture products, wheat flour samples spiked with the mixed AF standard solution (containing AFB_1_, AFB_2_, AFG_1_, and AFG_2_ with the concentrations of 200, 50, 200, and 50 ng mL^−1^, respectively) were simultaneously measured by ELISA and LC-MS/MS. Briefly, to 4 glass tubes containing 5 g of wheat flour, 0, 0.125, 0.25, and 0.5 mL of mixed AF standard solution were added, respectively, so that the AFB_1_ and AFG_1_ contents spiked in the tubes were 0, 5, 10, and 20 ng g^−1^, and the AFB_2_ and AFG_2_ contents were 0, 1.25, 2.5, and 5 ng g^−1^, (e.g., the total AF contents spiked in the tubes were 0, 12.5, 25, and 50 ng g^−1^, respectively) (Table 4). The samples were extracted with a methanol:water (8:2, *v*/*v*) solution, and subjected to a commercial immuno-affinity column (AflaOchraStar^TM^, Romer Labs, Getzersdorf, Austria) for clean-up. The elutes were measured by LC-MS/MS and ELISA.

### 3.9. LC-MS/MS Analysis

Chromatographic separation was performed on a Waters UPLC system (Waters Corporation, Product of Singapore) consisting of two pumps, a cooled autosampler, a column oven, and a column (Waters Acquity UPLC HSS T3, 100 mm × 2.1 mm I.D, 1.8 μm). Mobile phase A was Milli-Q water containing 0.1% formic acid water, and mobile phase B was methanol. A binary gradient system with a flow rate of 0.3 mL/min was established. The gradient steps were as follows: 5% B for 0–0.2 min, linear increase from 5 to 90% B for 0.2–4 min and holding for 6 min; 5% B for 6–8 min followed by re-equilibration of the column for 2 min.

A 5500 Triple Quad tandem mass spectrometer (AB Sciex, Framingham, MA, USA) equipped with a TurboIon Spray electrospray ion (ESI) source was used for detection. Sample introduction and ionization was conducted in the positive ion mode. The MS parameters were as follows: 5.5 kV ion spray voltage, 30 psi curtain gas pressure, 55 psi pressure for the nebulizer (gas1) and 65 psi pressure for the turbo (gas 2), 550 °C turbo heater temperature, 100 V declustering potential (DP). Collision energies of disparate analytes were optimized (AFB1: *m*/*z* 313.1–269, 40 eV; *m*/*z* 313.1–241.1, 47 eV; dwell time of 150 ms) using an automatic function of Analyst software 1.6.2 (AB Sciex). Data were acquired in multiple reaction monitoring (MRM) mode, and the scheduled MRM function was used as the acquisition method to ensure enough acquisition points (at least 12 points for each peak). The calibration curve for AFs was constructed with standards of 0.1, 0.5, 1.0, 2.0, 5.0, 8.0, and 10 ng mL^−1^.

## 4. Conclusions

A highly sensitive (at the pg mL^−1^ level) and group-specific ELISA using newly prepared mAb for the detection of AFB_1_ in the samples of agriculture and aquiculture products was described. The IC_50_ and LOD of the ELISA for AFB_1_ were 90 pg mL^−1^ and 18 pg mL^−1^, respectively, which were lower than those of most published ELISAs. There was no cross-reactivity (CR < 0.01%) of the assay with OTA, ZEN, DON, FB_1_, and PAT, but there was some extent of the CR values of the assay with AFB_1_ homologs, indicating the ELISA was able to evaluate total AF in agriculture samples. Accepted recoveries and RSD values of the ELISA for AFB_1_ in spiked agriculture and aquiculture samples were obtained. Wheat flour samples spiked with the mixed AF (AFB_1_, AFB_2_, AFG_1_, and AFG_2_) standard solution were measured by ELISA and LC-MS/MS simultaneously. It was demonstrated that the proposed ELISA was able to evaluate the total AF (AFB_1_, AFB_2_, AFG_1_, and AFG_2_) in wheat flour samples.

## Figures and Tables

**Table 1:** Comparison of published ELISAs with this work for AFB_1_ detection.

ELISA	Antibody	Sample	IC_50_ (ng mL^−1^)	LOD (ng mL^−1^)	Ref.
dc-ELISA	mAb	Rice	0.62	0.05	[20]
ic-ELISA	mAb	Food	0.33	/	[21]
ic-ELISA	pAb	Food	10	/	[22]
dc-ELISA	pAb	Feed	0.85	0.18	[23]
dc-ELISA	pAb	Corn, wheat, peanut	1.1	/	[24]
ic-ELISA	pAb	Chilli	1.2	/	[25]
ic-ELISA	mAb	Peanuts, corn, rice, etc.	0.13	/	[26]
dc-ELISA	pAb	Food, Feed	0.15	/	[27]
dc-ELISA	pAb	Egg	0.24	/	[28]
dc-ELISA	mAb	rice, pet food, peanut, etc.	0.051	/	[29]
dc-ELISA	mAb	Peanuts	0.09	/	[30]
ic-ELISA	mAb	Tea	0.057	/	[31]
dc-ELISA	pAb	Peanuts, Corn, etc.	0.8	/	[32]
ic-ELISA	pAb	Food	1.4	/	[33]
ic-ELISA	mAb	Wheat, corn, Crab roe	0.09	0.018	This work

**Table 2:** Cross-reactivity (CR) of the ELISA with six types of aflatoxins and five other types of fungal toxins.

Compound	Molecular Structure	IC_50_ (ng mL^−1^)	CR (%)
AFB_1_	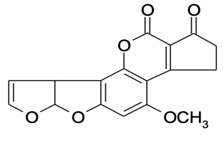	0.090	100
AFB_2_	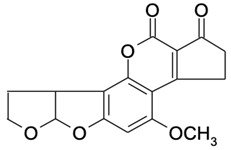	0.381	23.6
AFG_1_	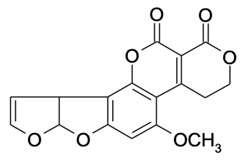	0.212	42.5
AFG_2_	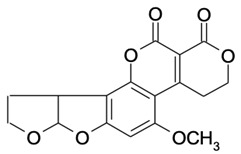	4.756	1.9
AFM_1_	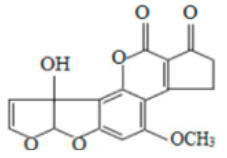	0.203	44.3
AFM_2_	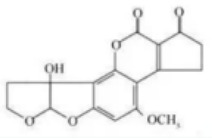	2.190	4.1
PAT	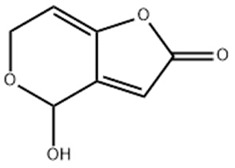	>1000	<0.01
FB1	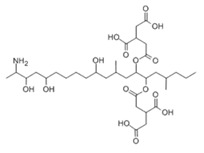	>1000	<0.01
ZEN	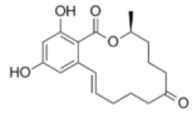	>1000	<0.01
DON	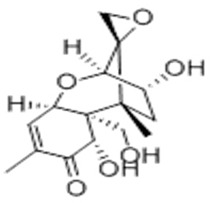	>1000	<0.01
OTA	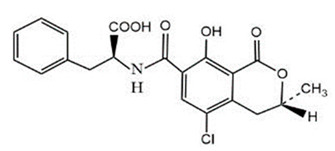	>1000	<0.01

**Table 3:** The recoveries of the AFB_1_ from spiked samples measured by ELISA.

Sample	Con. Spiked (ng g^−1^)	Con. Measured ±SD (ng g^−1^)	Recovery (%)	RSD (%, *n* = 3)
Wheat flour	0	0.90 ± 0.05	/	5.56
	2	3.21 ± 0.08	115.50	2.49
	5	6.73 ± 0.10	116.60	1.49
	10	11.27 ± 0.93	103.70	8.25
Corn flour	0	0.21 ± 0.01	/	4.76
	10	10.44 ± 0.75	102.30	7.18
	20	21.51 ± 0.32	106.50	1.49
	40	38.45 ± 0.62	95.60	1.61
Crab roe	0	0.37 ± 0.10	/	27.03
	10	8.20 ± 0.40	78.30	4.88
	20	20.67 ± 2.73	101.50	13.21
	40	37.67 ± 3.07	93.23	8.15

**Table 4:** Wheat flour sample spiked with mixed AFs measured by LC-MS/MS and ELISA.

Conc. of Total AFs Spiked (ng g^−1^)	Conc. of Total AFs Measured by LC-MS/MS (ng g^−1^)	Conc. Measured by ELISA (ng g^−1^)
0	2.46	0.25 ± 0.03
12.5	12.16	5.67 ± 0.33
25	23.21	12.73 ± 1.13
50	47.25	24.00 ± 2.80

## Data Availability

Data are contained within the article and Supplementary Materials.

## Supplementary Materials

The following supporting information can be downloaded at: https://www.mdpi.com/article/10.3390/molecules29102280/s1, Figure S1: Synthesis of AFB1–BSA conjugat; Table S1: Wheat flour sample spiked with AFs and measured by LC-MS/MS.
