# Supplementary material for: A Highly Sensitive and Group-Specific Enzyme-Linked Immunosorbent Assay (ELISA) for the Detection of AFB1 in Agriculture and Aquiculture Products
Source: Molecules. 2024 May 12;29(10):2280. doi: 10.3390/molecules29102280 (PMC11124235; doi:10.3390/molecules29102280)
Supplement: Supplementary file 1 [file molecules-29-02280-s001.zip › molecules-2975970-supplementary.pdf]

Supplemental materials

A highly sensitive and group-specific enzyme-linked immunosorbent assay (ELISA) for the detection of AFB_1 in agriculture and aquiculture products

Junlin Cao[a], Ting Wang[a], Kang Wu[b*], Fengjie Zhou[c], Yuze Feng[a], Jianguo Li[a,*], Anping Deng[a,*]

[a] College of Chemistry, Chemical Engineering & Materials Science, Soochow University, Renai Road 199, Suzhou 215123, China

[b] School of Biology & Basic Medical Science, Soochow University, Renai Road 199, Suzhou, 215123, China

[c] Suzhou Agricultural Products Safety and Quality Inspection Center, Wuzhong Road 1399, Suzhou, 215000, China

* Corresponding authors: Tel: +86 512 65884630; Fax: +86 512 65884630
e-mail: wukang650202@163.com; lijgsd@suda.edu.cn; denganping@suda.edu.cn

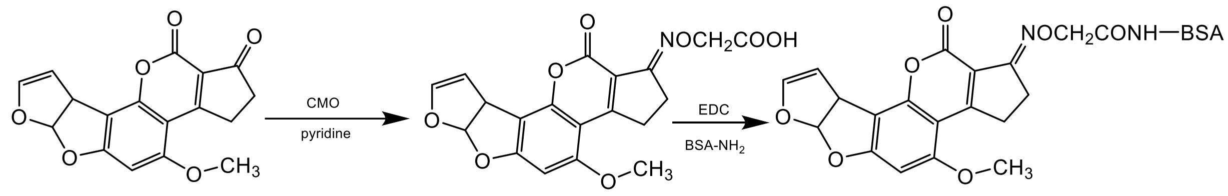

Fig. S1. Synthesis of AFB_1–BSA conjugate

Table S1. Wheat flour sample spiked with AFs and measured by LC-MS/MS

AFB_1		AFB_2		AFG_1		AFG_2	
Conc. Spiked (ng g^{-1})	Conc. Measured (ng g^{-1})	Conc. Spiked (ng g^{-1})	Conc. Measured (ng g^{-1})	Conc. Spiked (ng g^{-1})	Conc. Measured (ng g^{-1})	Conc. Spiked (ng g^{-1})	Conc. Measured (ng g^{-1})
0	1.40	0	0.21	0	0.75	0	0.09
5	5.55	1.25	0.96	5	4.63	1.25	1.03
10	9.68	2.5	1.80	10	9.57	2.5	2.17
20	20.11	5	3.70	20	19.13	5	4.30
